# Transcriptome Analysis Reveals Genes Associated With Sexual Dichromatism of Head Feather Color in Mallard

**DOI:** 10.3389/fgene.2021.627974

**Published:** 2021-12-08

**Authors:** Shengchao Ma, Hehe Liu, Jianmei Wang, Lei Wang, Yang Xi, Yisi Liu, Qian Xu, Jiwei Hu, Chunchun Han, Lili Bai, Liang Li, Jiwen Wang

**Affiliations:** Farm Animal Genetic Resources Exploration and Innovation Key Laboratory of Sichuan Province, Sichuan Agricultural University, Chengdu, China

**Keywords:** mallards, sexual dimorphism, feather color, TYR, TYRP1, Z-chromosome

## Abstract

Sexual dimorphism of feather color is typical in mallards, in which drakes exhibit green head feathers, while females show dull head feather color. We showed that more melanosomes deposited in the males’ head’s feather barbules than females and further form a two-dimensional hexagonal lattice, which conferred the green feather coloration of drakes. Additionally, transcriptome analysis revealed that some essential melanin biosynthesis genes were highly expressed in feather follicles during the development of green feathers, contributing to melanin deposition. We further identified 18 candidate differentially expressed genes, which may affect the sharp color differences between the males’ head feathers, back feathers, and the females’ head feathers. *TYR* and *TYRP1* genes are associated with melanin biosynthesis directly. Their expressions in the males’ head feather follicles were significantly higher than those in the back feather follicles and females’ head feather follicles. Most clearly, the expression of *TYRP1* was 256 and 32 times higher in the head follicles of males than in those of the female head and the male back, respectively. Hence, *TYR* and *TYRP1* are probably the most critical candidate genes in DEGs. They may affect the sexual dimorphism of head feather color by *cis*-regulation of some transcription factors and the Z-chromosome dosage effect.

## Introduction

Plumage color differences between males and females are common in most avian species, such as Anna’s hummingbird, chicken, Japanese quail, mallard, turkey, and zebra finch (Zuk et al., 1990; Collins and Cate, 1996; Zann 1996). The nuptial plumage of adult males is beautiful and colorful, but the plumage color of females is dull. These characteristics are known as sexual dimorphism in avian species (Andersson 1994). Since Darwin, the function and evolution of sexual dimorphism in plumage color have been a popular field among evolutionary biologists (Darwin 1888). Some studies have shown that sexually dichromatic species have evolved from sexually monochromatic ancestors through sexual selection for sexually dichromatic trait elaboration (Andersson 1994). In the long process of sexual selection, the male’s nuptial feathers give the carrier a mating advantage (Slagsvold and Lifjeld 1992). Models of sexual selection have suggested that the feather color characteristics of males are flexible, reflecting the viability and body condition of the carrier (Zahavi 1975; Andersson 1986; Grafen 1990).

Mallard (*Anas platyrhynchos*) is one of the best models for studying feather color sexual dimorphism. The drake has metallic green feathers on its head, a white ring under its neck, and brownish gray and stripes of feathers on the back, whereas the female duck has dark brown feathers on the head and back. The development of the metallic green (head) and blue (wings) coloration of mallards is very complex, as it is directly related to pigmentation deposition inside feathers. The pigments of organisms can be mainly divided into two categories: carotenoids and melanins. Carotenoids can absorb light waves with wavelengths ranging from blue to green, which causes feathers to exhibit yellow, orange, or red coloration (McGraw and Hill, 2006). Melanins are more widely distributed and can be divided into eumelanin and phaeomelanin, which cause gray to black feathers and golden to rusty red feathers, respectively (Stavenga and Wilts 2014). When these two kinds of melanin are mixed, an intermediate color is produced (Hill 2010). Furthermore, these colors are related to coherent light scattering caused by nanostructure inside the feathers (Prum 2006). This ordered nanostructure is originated from the assembly of melanosomes during feather development. This structure is analogous to a two-dimensional hexagonal lattice (Durrer 1997; Stavenga et al., 2017). The nuptial plumage color is neutral coloration in drakes, and the development of this neutral color is related to regulation by sex hormones (Witschi, 1961; Ralph 1969). Estrogen inhibits the growth of nuptial plumage in female ducks. The effect of testosterone is similar to that of estrogen in drakes (Haase et al., 1992). Sex hormones may affect the biosynthesis or transfer of melanin in cells.

Numerous functional genes determining feather color have been identified in avians. Most of these genes regulate the color of feathers by affecting melanocyte migration or melanin biosynthesis (Zhou et al., 2018; Li et al., 2012). Although, some studies have explained some mechanisms, such as environmental factors or carotenoid levels, which could cause sexual dimorphism in plumage color in avian species (Hill 2000; Johnsen et al., 2003; Hubbard et al., 2015), and Gazda *et al.* have shown that sexual dimorphism of some avian species is determined by carotenoid-cleaving enzyme β-carotene oxygenase 2 (*BCO2*) (Gazda et al., 2020). However, few reports have revealed the genetic mechanism of melanin-based sexual dimorphism in avian species.

**FIGURE 1:**
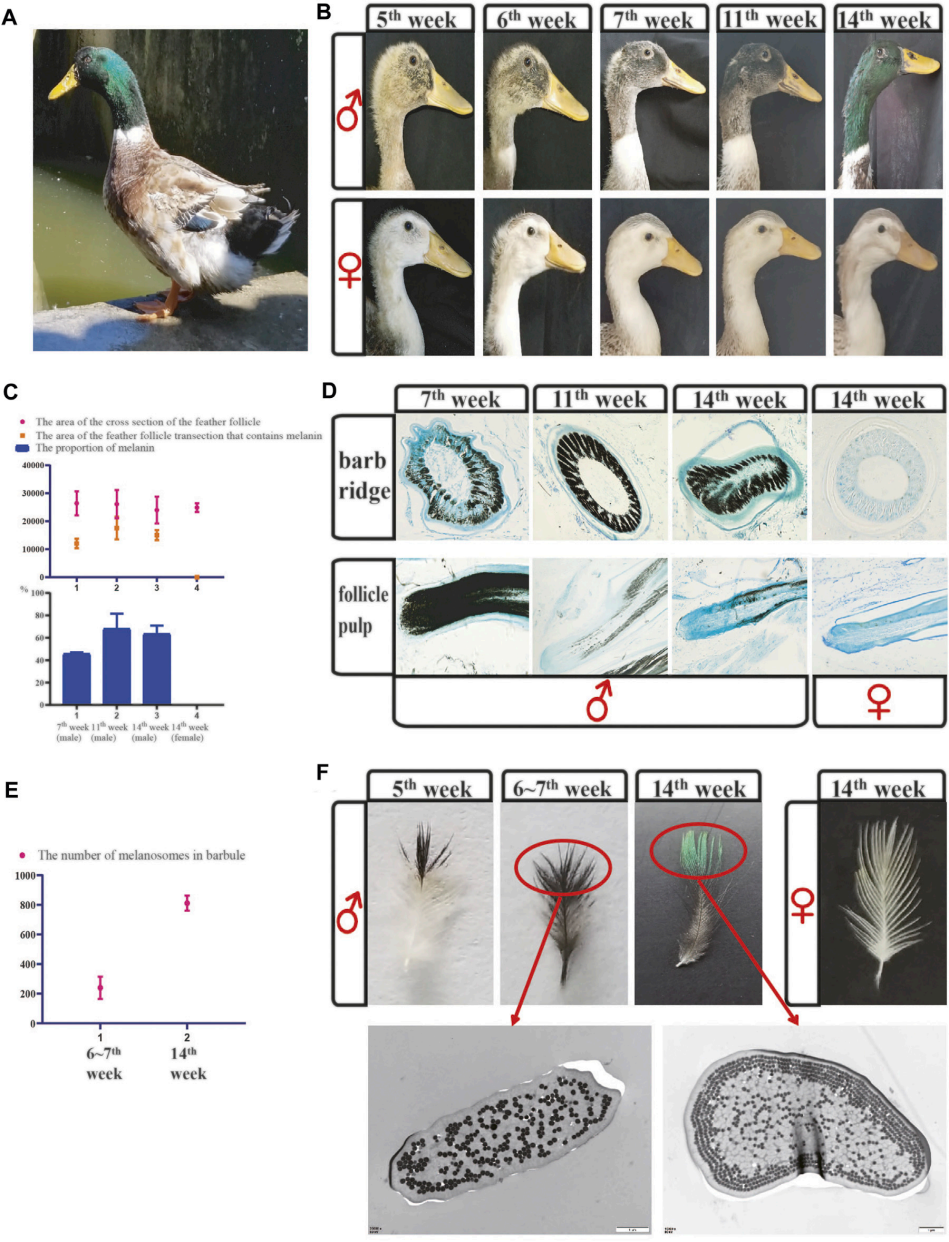
The difference in feather color between males and females revealed microscopic and anatomical observations. **(A)** Image of a male mallard. **(B)** Color changes of the head feathers in mallards at different growth stages. **(C)** The melanin content in the barb ridges of mallards was measured using IPP software. **(D)** Cross-sections of the barb ridges and vertical sections of the feather follicle pulp in mallards at different growth stages. **(E)** The number of melanosomes was calculated using IPP software in the barbules of mallards. **(F)** In Figure 1F, the feathers were isolated from the head of male and female mallards at different growth stages. And the Figure 1F shows TEM sections of individual small barbules from the distal region indicated TEM (transmission electron microscope) images of vertical sections of the small barbules; scale bar = 1 μm.

We used mallard (*Anas platyrhynchos*), (Figure 1A) to investigate the genetic basis of green feather development in the drake head. First of all, we observed the development of the feathers on the mallard’s head. Next, we investigated the anatomical characteristics of the coloration of the head feathers and feather follicles of mallards during the head feather formation process of the drakes. Then we further investigated the genome-wide expression of the head and back feather follicles by RNA-Seq during these periods. Finally, we compared the transcriptome differences in the male head vs. female head, male head vs. male back, and male head in the 7th week *vs.* male head in the 11th week, and the genes related to green plumage color were screened. Our work reveals the association between gene sex-biased expression and melanin deposition in feather follicles of mallard for the first time. It provides a new idea and reference for the regulatory study of melanin biosynthesis in melanocytes of other species. Meanwhile, our findings may provide a reference illustrating the mechanism of sexual dimorphism in the plumage color of avians.

## Materials and Methods

### Animals and Sampling

The fertilized eggs of mallards were collected from the waterfowl breeding farm of Sichuan Agricultural University, Ya’an. After incubation, the hatched mallards were maintained for 14 weeks in a comfortable environment. These ducks could freely feed and drink throughout their growth stages. The head and back skin tissue of drakes and the head skin tissue of females were sampled at the ages of 7, 11, and 14 weeks. We considered the difficulty of sampling feather follicles, the feather follicles to stay in the skin, and tested the skin tissue directly. An equal amount of skin tissue in each sample and feather follicles remained in each skin sample. In addition, feather samples were collected from 6- to 7-week-old and 14-week-old mallards. After sample collection was completed, some of the skin tissue samples were stored at −80°C, and the remaining skin tissue samples were stored in 4% paraformaldehyde, while the feather samples were stored in 3% glutaraldehyde.

### Light Microscopy Observations

The skin samples were dehydrated with EthOH in successive concentrations of 70, 80, 90, 95, 99, and 100%. The samples were further infiltrated three times in xylene. Each dehydration step was performed in a dehydration system (Leica TM 1020, Germany) for 22 h, and the tissues were then paraffin-embedded. We investigated skin anatomy in cross-sections and vertical sections. The skin tissue samples were cut using microtome blades on a Leica RM 2135 microtome. The sections were stained with L-Dopa (Solarbio, China). Images of the tissue sections were obtained on an Olympus SZX16 stereomicroscope (Japan). Finally, the melanin content of the barb ridges was analyzed using IPP 6.0 (Image-Pro Plus 6.0). The melanin content was expressed by calculating the area occupied by the melanin, which was stained dopa in the cross-section of the feather follicles.

### Transmission Electron Microscopy Observations

Barbule anatomy was investigated by transmission electron microscopy (TEM) using standard methods (Thorpe et al., 2008). Briefly, a feather piece was subjected to a series of dehydration in acetone at increasing concentrations of 30, 50, 70, 80, 90, 95, and 100% and then infiltrated with epoxy resin at 3:1, 1:1, and 1:3 proportions. Next, we cured the resin blocks by heating them and cut them into approximately 50 nm sections using a Leica EM UC7 ultramicrotome (Leica Microsystems GmbH, Germany). The sections were stained using uranyl acetate and lead citrate. Next, we checked the sections on a JEM-1400PLUS TEM (Japan). Finally, the number of melanosomes was analyzed by using IPP 6.0.

### RNA Isolation and RNA-Seq

Total RNA was extracted from duck skin tissues using TRIzol (RNAiso Plus, Takara, Japan). Illumina platform sequencing of all samples was conducted at Novogene Bioinformatics Technology Co., Ltd., Beijing. In total, 15 sample libraries were constructed, including 12 libraries from head skin samples (three repeats from each stage of drake sampling in the 7^th^, 11th^,^ and 14th weeks and three repeats from female mallards sampled in the 14th week) and three from dorsum skin samples collected from drakes in the 14th week. Low-quality reads (i.e., tags containing only adaptors and ambiguous bases) were removed from all samples.

### Bioinformatics Analysis

HISAT 2.0 software (Kim et al., 2015) was used to map the sequencing data with the assembly of the mallard reference genome (IASCAAS_PekingDuck_PBH1.5, GCF_003850225.1) to generate read count results. Second, we used STEM (Short time-series expression miner) software to analyze the genome-wide expression trends in the partial FPKM results, including three growth stages (7th weeks, 11th weeks, and 14th weeks) of drakes. Third, differential gene expression was tested using DESeq 2.0 from the R package (Love et al., 2014). The log_2_ (fold-change) values of the genes were tested using DESeq to evaluate the expression patterns based on all sequencing data. Fourth, the gene ontology (GO) pathways of differentially expressed genes were analyzed using the R package’s cluster profile (Yu et al., 2012). Finally, we used GraphPad 8.0, BioVenn, and MeV 4.9 software to generate the volcano plots, Venn diagram, and heat map of gene expression, respectively.

### Transcription Factor Prediction

The online transcription factor (TF) binding site prediction tool (http://bioinfo.life.hust.edu.cn/AnimalTFDB/#!/tfbs_predict) from the Animal TFBD database was used to predict the TF binding sites in the promoter regions of the *TYR* (Chr1- 8,264,483∼82,694,830 bp) and *TYRP1* genes (ChrZ- 307,517,598∼30,752,598 bp).

### Synteny Analysis

In the synteny analysis, we analyzed six avian assemblies from NCBI (National Center for Biotechnology Information), including assemblies for mallard (IASCAAS_PekingDuck_PBH1.5), Anna’s hummingbird (bCalAnn1_v1.p, GCF_003957555.1), chicken (GRCg6a, GCF_000002315.6), Japanese quail (*Coturnix japonica* 2.1, GCF_001577835.2), turkey (Turkey 5.1, GCF_000146,605.3), and zebra finch (bTaeGut1_v1.p, GCF_003957565.1). These six species are representative avian species, and their genomes have been well annotated.

### Phylogenetic Analysis

We obtained the coding regions (CDs) of five genes (*TYRP1*, *PTPRD*, *LURAP1L*, *MPDZ*, and *NFIB*) of 6 avian species from the NCBI database (Supplementary Table S1). We used Clustal Omega of MEGA 7.0 (Sudhir et al., 2016) to perform multisequence alignment of these sequences. Revised sequence alignments were submitted to MEGA 7.0 to construct a bootstrap (1000 replicate) tree (Felsenstein 1985) for each gene. The maximum likelihood (ML) method was used for phylogenetic analysis. We identified the best DNA models for each gene (*TYRP1*: K2 + G, *PTPRD*: T92 + G, *LURAP1L*: TN93 + G, *MPDZ*: K2 + I, and *NFIB*: K2). The ML search started with the tree generated by BioNJ (Make initial tree automatically) (Gascuel 1997), and the optimal tree was determined via a heuristic search using the NNI (nearest-neighbor interchange) algorithm.

### Data Statistics

We performed all statistical analyses using R packages (R Core Team, 2014). A *p*-value < 0.01 was considered highly significant, while a *p*-value < 0.05 was considered statistically significant.

## Results

### Anatomy of Follicles and Nanostructures of Feather Barbules

The villi on the heads of the mallard ducklings began to transform into contour feathers at the age of 5 or 6 weeks. Initially, new feathers grew in areas near the eyes of drakes (Figure 1B). Then, at the 6th to 14th weeks, new feathers gradually developed in the head and neck (Figure 1B). At the 14th week, the feathers finally turned green and covered the drakes’ heads entirely (Figure 1B). Throughout the growth period of female mallards, the feathers in most areas were light in color (Figure 1B) and did not change further.

We performed histological observations to obtain a better understanding of green feather development. In short, feathers develop from the feather follicles. The feather follicles consist of the dermal papilla and epidermal collar, and the proliferation of the epithelial cells is active at the bottom of the feather follicles. During the development of the feathers, epithelial cells differentiate to the barb ridges at the top of the feather follicles firstly. And the barb ridges further proliferates and differentiates into the marginal plates, barbule plates, and axial plates, and finally form the mature feathers. During this process, mature melanocytes located in keratinocytes release the melanosomes. And the melanosomes transfer from the bottom of the feather follicles to the small barbules (Yu et al., 2004). In the barb ridges of mallard drakes (Figure 1D), melanin began to deposit at the age of 7 weeks. The melanin content increased significantly at the ages of 11th and 14th weeks (Figures 1C,D). In female ducks, melanin deposition was not observed at 14 weeks (Figures 1C,D).

According to the observations of the vertical sections of the follicle pulp of males (Figure 1D), melanin mainly transferred from the dermal papilla to the barb ridges. At the age of 7 weeks, intense melanin deposition was observed in the follicle pulp. At the age of 14 weeks, less melanin was observed in follicle pulp areas. However, there was still less melanin in females’ follicle pulp areas (Figure 1D).

In the feathers, only the barbules at the top showed black coloration at the duck age of 5 weeks (Figure 1F), which gradually changed from black to green at the ages of 6–14 weeks. We further investigated cross-sections of the small barbules between the black parts of feathers in the 6th∼7th weeks and the green parts of feathers in the 14th week (Figures 1E,F). There were few melanosomes in the barbules of the black regions, and they were irregularly arranged under the keratin layer. In contrast, in the barbules of the green parts, there were more melanosomes located under and enwrapped by the keratin layer, and they were arranged in an orderly pattern (4 layers) (Figures 1E,F). Taken together, we suggested that the development of green feathers may be caused by the abundant melanin transferred to the small barbules. We further assumed that the excessive deposition of melanin/melanosomes caused the orderly arrangement of melanosomes and eventually led to green feather development through coherent emitted light scattering.

### Overview of RNA-Seq Data

A summary of the sequencing reads and matched genes is shown in Supplementary Table S2. After the low-quality reads were removed, an average of 5,000,000 clean reads remained for each sample. In addition, an average of 93.2% reads was mapped to the reference genome of mallard (IASCAAS_PekingDuck_PBH1.5, GCF_003850225.1).

### Gene Expression Profile Clusters During Green Feather Development

**FIGURE 2:**
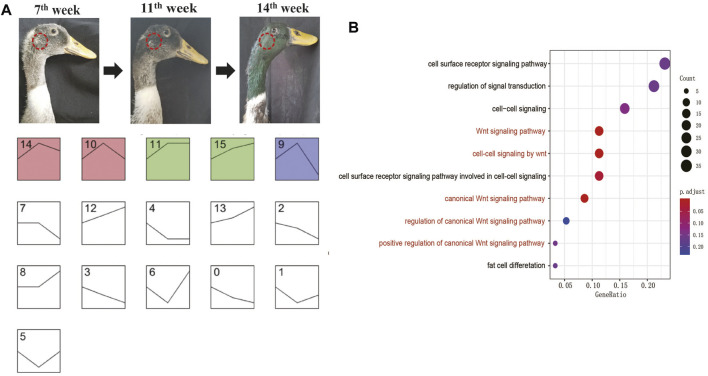
Genome-wide gene expression trend analysis. **(A)** Classification of genome-wide gene expression trends in three growth stages. The sampling position is marked on the mallard. Each simplified line chart shows a trend of gene expression. The *Y*-axis coordinates of the line graph are the FPKM value, and the *X*-axis coordinates are the growth stages (The three nodal points in the line graph represent 7th, 11th, and 14th weeks from left to right). **(B)** GO analysis of the genes in category 10.

To understand the genes involved in developing green feather color on the drake head, we performed gene expression profile cluster analysis based on the transcriptome data obtained at the 7th week, 11th week, and 14th week during green head feather development. The results showed that all genes could be divided into 16 categories (0–15), among which five significant types were found, including 14, 10, 11, 15, and 9. Furthermore, among the five categories, the expression patterns of categories 14 and 10 and those of categories 11 and 15 were similar (Figure 2A).

In these five significant categories, the expressions of all genes were upregulated in the 7th–11th weeks and downregulated in the 11th–14th weeks. In category 10, there were 16 genes whose functions were related to melanocyte development (*EDNRB*, *KIT*, *LYST*, *SOX10*), melanin production (*POMC*, *OCA2*, *TYR*, *TYRP1*, *TRPM1*, *SLC45A2*, *SLC24A4*, *PAH*), and the melanin distribution (*MYO5A*, *RAB27B*, *MLANA*) (Jackson 1994; Bellone 2010; Cieslak et al., 2011; Sturm and Duffy, 2012). Two other genes in category nine were related to melanin production (*MC1R, RAF1*) (Jackson 1994; Bellone 2010; Cieslak et al., 2011; Sturm and Duffy, 2012), while three genes in category 14 were related to melanin production (*CLCN7*, *HPGDS*, *STX17*) (Jackson 1994; Bellone 2010; Cieslak et al., 2011; Sturm and Duffy, 2012). GO analysis further showed that (Figure 2B; Supplementary Table S3) signaling pathways associated with BMP and Wnt were significantly enriched. These signaling pathways were reported to play roles in melanin biosynthesis (Huang et al., 2011) and feather development (Rouzankina et al., 2004). Hence, the transcriptomic analysis revealed that most pigmentation genes were highly expressed in feather follicles during green head feathers, contributing to melanin deposition. Meanwhile, the results also showed that 7–11 weeks might be a more rapid period for melanin biosynthesis than 11–14 weeks in the males’ heads.

### The Causative Genes Determining Male Green Feathers

**FIGURE 3:**
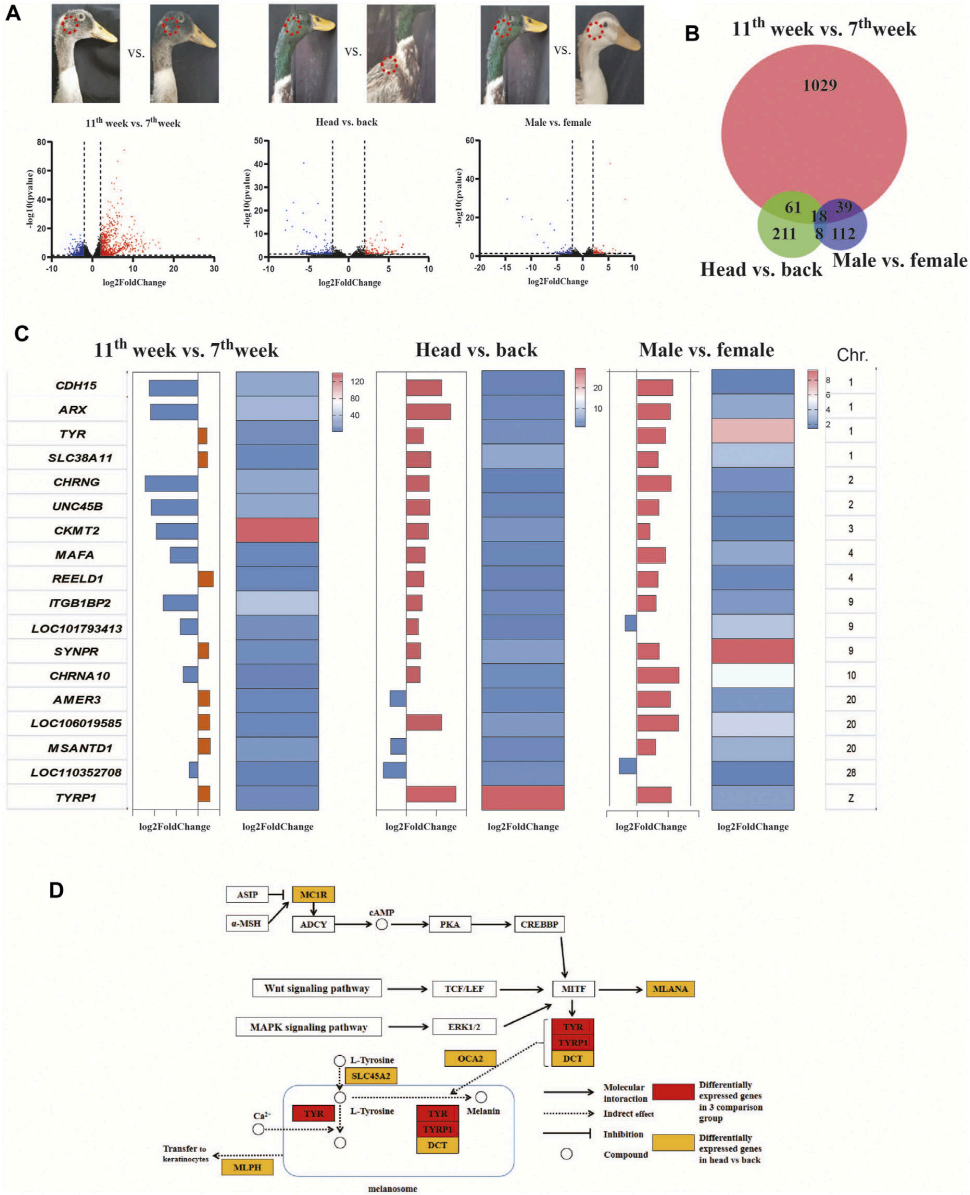
Screening of the causative genes of the male green head feathers. **(A)** Volcano plots showing the differentially expressed genes (DEGs) of 3 comparison pairs: 11 w *vs.* seven w, male *vs.* female, and head *vs.* back. We have indicated the sampling points in each image of a mallard. **(B)** The Venn diagram shows the number of DEGs among the three comparison groups. **(C)** The consensus genes were differentially expressed among the three comparison groups. **(D)** The signaling pathway of melanin biosynthesis.

To screen causative genes, we screened the differentially expressed genes (DEGs) among three comparisons: male head *vs.* female head, male head *vs.* male back, and male head in the 7th week *vs.* male head in the 11th week. The results showed that 1147, 297, and 177 DEGs were identified (Figure 3A). GO analysis of the DEGs further revealed that the *TYR* and *TYRP1* genes were enriched in the pigment biosynthesis process, pigmentation, pigment metabolic, melanosome, and pigment granule pathways (Supplementary Tables S4–S6).

The candidate genes responsible for the male green head feathers should explain the differences between all green and nongreen feather samples; thus, we checked the consensus DEGs among the three comparison pairs. Venn analysis identified 18 genes as potential causative genes determining green male head feathers (Figure 3B,C). Among these genes, only *TYR* and *TYRP1* were involved in melanin biosynthesis, which are critical downstream genes of the three melanin synthesis pathways (cAMP, Wnt, and MAPK pathways) (Figure 3C). In contrast, others were related to receptor proteins, molecular binding, and cellular structure. In the three comparison groups, the expression levels of *TYR*, *SLC38A11*, *REELD1*, *SYNPR*, *LOC106019585*, and *TYRP1* were all up-regulated in green samples, while only the *LOC110352708* was down-regulated (Figure 3D). In comparing males vs. females, the TYRP1 and TYR gene expression were 256 and 32 times higher in the head feather follicles of males than in females’ head feather follicles and the males’ back feathers, respectively. Among the 18 candidate genes, *TYRP1* was located on the Z-chromosome, and the other genes (including *TYR*) were located on euchromosomes.

**FIGURE 4:**
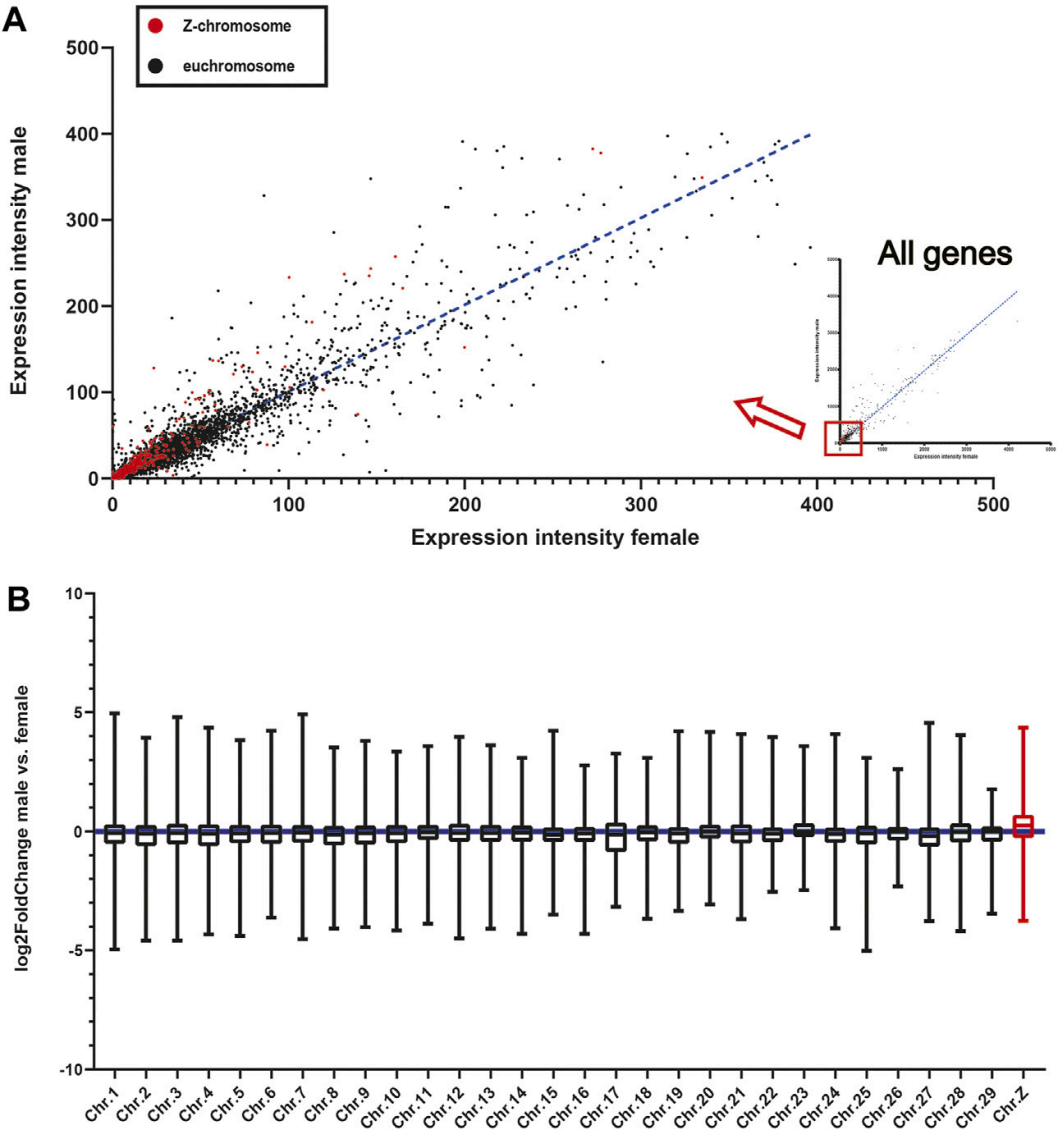
The expression patterns of Z-chromosome-linked and Autosome-linked genes in mallards. **(A)** The expression levels of individual Z-chromosome-linked and euchromosome-linked genes. Each point in the scatter diagram represents the FPKM value of a gene in the male and female head samples. **(B)** Box plots showing the median log_2_-fold change values per chromosome of male vs. female. The whiskers extend to the minimum and maximum values without outliers (a log_2_ fold-change defines outliers > five or < −5).

Finally, as males in birds have two Z-chromosomes while females only have one, we compared the gene expression patterns of the head feather follicles of male and female mallards. First, we found that the expression levels of most Z-chromosome-linked genes (Accounted for about 63% of the expressed genes) were higher in males than in females (Figure 4A; Table S11). Then, we analyzed the average log_2_ fold-change values of all genes on each chromosome, and we found that the Z-chromosome genes exhibited higher log_2_ fold-changes than those on all euchromosomes (Figure 4B). Therefore, we believed it is one reason that the dosage effect of the Z-chromosome in males may lead to higher expression of the *TYRP1* gene in male head feather follicles than in females.

### The *Cis*-regulation of the *TYRP1* Gene May Explain Why Only Head Feathers Are Green

**FIGURE 5:**
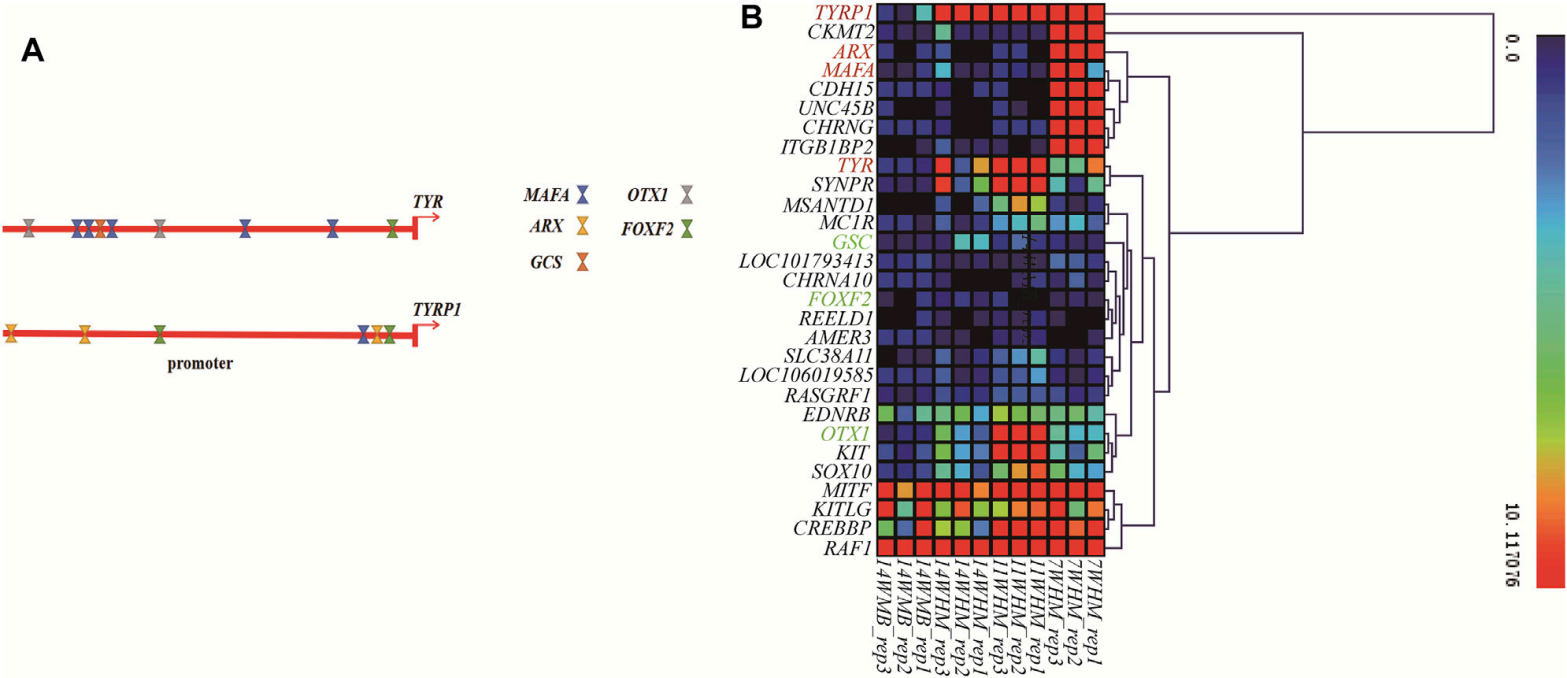
The effect of transcription factors on *TYR* and *TYRP1* gene expression. **(A)** The TF binding sites of the *TYR* and *TYRP1* promoters were predicted. **(B)** Some pigmentation genes and 18 causative genes were expressed in 12 male samples. We used the FPKM results for the above samples for heat map construction.

The transcription factors (TFs) binding sites of the duck *TYR* and *TYRP1* genes were predicted using online prediction software with the promoters as input. All predicted TFs are provided in Supplementary Tables S7, S8. Binding sites of MAFA (MAF bZIP transcription factor A), GSC (goosecoid homeobox), OTX1 (orthodenticle homeobox 1), and FOXF2 (forkhead box F2) TFs were predicted in the *TYR* promoter region. In contrast, binding sites of MAFA, ARX (aristaless related homeobox), and FOXF2 were predicted in the *TYRP1* promoter region (Figure 5A). Among these TFs coding genes, *MAFA* and *ARX* are members of 18 potential causative genes in the three comparison groups At the same time, *GSC*, *OTX1*, and *FOXF2* were differentially expressed only between the head follicles and back follicles of drakes (Supplementary Table S9). Based on gene expression cluster analysis (Figure 5B) of the drake samples, we observed that ARX and MAFA expression patterns were most similar to those of the *TYRP1* gene. Thus, the differential expression of *TYRP1* between different body parts and time points in males was possibly due to differences in the *cis*-regulation of potential transcription factors.

### Synteny and Phylogenetic of the Z Chromosome-Linked Pigmentation Genes Between the Mallard and Other Avian Species

**FIGURE 6:**
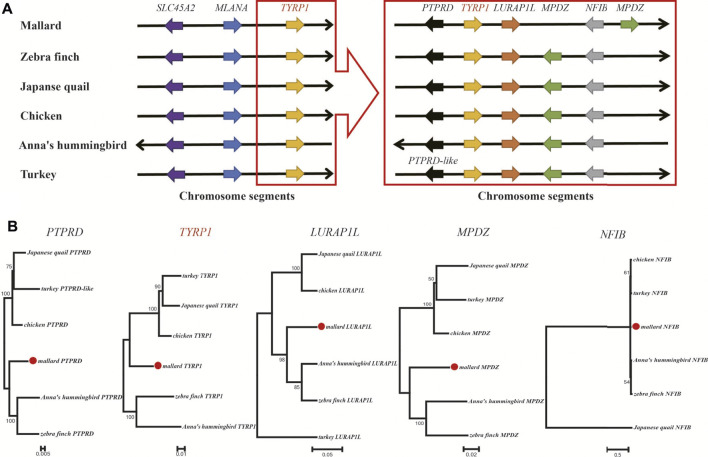
Synteny and phylogenetic analysis of *TYRP1* and its neighboring genes in avians. **(A)** The relative positions of pigmentation genes and the adjacent genes of *TYRP1* in Z-chromosome segments. Each arrow color represents a gene. We arranged the order of the arrows based on the relative gene positions on the chromosome. **(B)** Phylogenetic trees of five neighboring genes of the *TYRP1* gene.

Sexual dimorphism in plumage color is common in avian species. Therefore, we further checked the pigmentation genes on the Z-chromosomes of six avian species showing sexual dimorphism of plumage color characteristics. The genomes of these six avian species were well assembled and annotated. Three pigmentation genes, *MLANA*, *SLC45A2*, and *TYRP1*, are located on the Z-chromosome (Supplementary Table S10). Synteny analysis (Figure 6) showed that the *TYRP1* gene and its neighboring genes were positioned according to their relative locations on the Z-chromosome. Four genes adjacent to *TYRP1*, including *PTPRD*, *LURAP1L*, *MPDZ*, and *NFIB*, were conserved in the six avian species and shared a similar transcription direction. Phylogenetic analysis (Figure 6) further suggested that the above five genes presented similar phylogenetic relationships among the six avian species. These findings implied that the chromosomal segment carrying the *TYRP1* gene and its neighboring genes were conserved in avian species.

## Discussion

The sexual dichromatism of plumage color in avian species helps us understand sexual and natural selection and their roles in speciation. In avian species, females prefer bright plumage to males, which gives the more colorful males a mating advantage (Slagsvold and Lifjeld 1992). In our work, we used mallards as a model to study the mechanism underlying the development of sexual dichromatism in plumage color.

As a characteristic of sexual dichromatism, the green feathers on the drake’s head contrast sharply with the female’s dull feathers. Previous studies have suggested that multiple mechanisms determine plumage color (Hill 2000; Doucet 2009; Maia et al., 2009; Prum et al., 2009; Maia et al., 2012). Among these mechanisms, pigmentation is necessary for feather coloration. Our anatomical observations showed that melanin was continuously deposited in the head feather follicles during drake development. However, in the head feather follicles of females, melanin deposition was not observed at the age of 14th weeks. Haase *et al.* investigated the melanin contents of the feather follicles of male and female mallards by HPLC (High-performance liquid chromatography). They found that the eumelanin content in the head feather follicles of drakes was significantly higher than in the feather follicles of other body parts and the female head, which was consistent with our results. After melanin was transferred from the feather follicles to the feather barbules on the drake head, the melanin/melanosomes were further arranged in an orderly pattern under the keratin layer of the feather barbules. Our results were comparable with the results of Stavenga et al., 2017 and Eliason and Shawkey (2012). Additionally, Eliasond *et al.* and Khudiyev et al. (2014) found that this ordered arrangement was similar to a two-dimensional hexagonal lattice and caused the feathers to appear green by scattering light. The two-dimensional hexagonal lattice may be highly variable, allowing color to be altered by minor variations in lattice constants, the melanosomal radius, and cortical thickness. Based on the previous conclusions and our results, we can be sure that the difference of melanin deposition between males’ and females’ head feather follicles causes the sexual dichromatism of feather color directly.

Additionally, transcriptome data revealed that the expression of melanin biosynthesis genes began to increase during drake development and eventually remained high. These genes are mainly involved in the Wnt signaling pathway, MAPK signaling pathway, melanosome formation, and precursor transport. Collectively, these findings indicated that the development of green feathers was related to the excessive deposition and ordered arrangement of melanin in feather barbules.

We further revealed the underlying genes that affect the sexual dimorphism of feather color in mallards. First, we identified 18 consensus candidate DEGs, which may cause the sharp color differences between the males’ head feathers, back feathers, and the females’ head feathers. Among these genes, *TYR* and *TYRP1* were involved in melanin biosynthesis. Tyrosinase (TYR) and tyrosinase-related protein 1 (TYRP1) regulates the development of 5,6-dihydroxyindoleic acid (DHICA), which is the precursor of eumelanin (Wang et al., 2017 and Olivares et al., 2001). Notably, the expression of the *TYRP1* gene was significantly different in different body parts (head *vs.* back) and between the sexes (male head *vs.* female head) than other candidate genes. Therefore, we believe that the *TYR* and *TYRP1* gene is the primary candidate genes, which determine the green feathers of males. Xu *et al.* also showed that melanic plumage color in Korean quails was associated with either increased production of *TYR* or decreased production of *TYRP1* (Xu et al., 2013). These studies also demonstrated the importance of the *TYRP1* gene for feather color regulation.

Next, we studied the factors affecting the *TYR* or *TYRP1* gene expression. The following two factors may affect the *TYR* or *TYRP1* gene expression: 1) the Z-chromosome dosage effect and 2) the *cis*-regulation of some TFs. The two most common forms of chromosomal sex determination are the male heterogametic XX/XY system and the ZZ/ZW system, and females are the isogamete and heterogametic sex in XX/XY and ZZ/ZW system, respectively. In mammals this is achieved by one of the X-chromosomes inactivation and in flies and worms by up- or down-regulation of X-linked expression (dosage compensation), respectively (Brockdorff and Turner 2015). Unlike mammals and insects, when male avian acquired more Z-chromosomes than females, the females of the avian species did not exhibit compensation. Thus, the expression levels of Z-chromosome-linked genes in male avian species is higher than that in female avian species (dosage effect) (Wolf and Bryk 2011; Mank 2013). Itoh *et al.* found that males exhibited up 40% higher expression of Z-chromosome-linked genes than the females in zebra finch (Itoh et al., 2007). We found that the Z-chromosome dosage effect also existed in mallards based on transcriptome analysis. This suggested that the two allelic *TYRP1* genes may be expressed simultaneously in drake head feather follicles. hence, the expression of *TYRP1* may be influenced by Z-chromosome dosage effect. Additionally, at least three melanin biosynthesis genes, including the *TYRP1* gene, were located on the Z-chromosome of mallards and the other five other avian species. Then, we also found that the Z-chromosome segment containing the *TYRP1* gene was conserved in mallards and five other avian species.

Eukaryotic transcription initiation is complex and requires the involvement of many transcription factors. According to the results of TFs prediction and gene expression cluster analysis, the expression patterns of five transcription factors (*MAFA*, *ARX*, *OTX1*, *GSC*, and *FOXF2*) were similar to that of the *TYR* and *TYRP1* gene, suggesting that transcription factors mainly caused the differential expression of *TYR* and *TYRP1* among body parts (head vs. back) in drakes and different sexes. We presumed that the color difference between back and head may be caused by the differential expression of *TYR* or *TYRP1* genes in drake. Meanwhile, we also consider that the differential expression of *TYR* or *TYRP1* among different body parts (head *vs.* back) in drakes may be mainly caused by some TFs. However, the specific regulatory mechanism of these candidate genes remains to be further studied.

## Conclusion

More melanosomes are deposited in the males’ head’s feather barbules than females, which may cause the sexual dimorphism of head feather color in mallards directly. In addition, some melanin biosynthesis genes that were highly expressed may contribute to the development of the green feathers. In 18 candidate genes, *TYR* and *TYRP1* are probably the most critical genes affecting the sexual dimorphism of head feathers in mallards. Meanwhile, the expression of *TYR* or *TYRP1* may also be influenced by some transcription factors and the Z-chromosome dosage effect.

## Data Availability

The transcriptome datasets are available in the SRA (BioProject ID: PRJNA679685).
