# Supplementary material for: Transcriptome Analysis Reveals Genes Associated With Sexual Dichromatism of Head Feather Color in Mallard
Source: Front Genet. 2021 Dec 8;12:627974. doi: 10.3389/fgene.2021.627974 (PMC8692775; doi:10.3389/fgene.2021.627974)
Supplement: Supplementary file 2 [file Table1.DOCX]

**Table S1. The genebank numbers of five genes in phylogenetic analysis.**

| **Gene name** | **mallard** | **chicken** | **turkey** | **Japanese quail** | **zebra finch** | **Anna's hummingbird** |
| --- | --- | --- | --- | --- | --- | --- |
| ***TYRP1*** | XM_027447040.1 | NM_205045.2 | XM_010725651.2 | XM_015848997.2 | XM_030258518.2 | XM_030466744.1 |
| ***PTPRD*** | XM_027446713.1 | XM_025145217.1 | XM_031557282.1 | XM_015848997.2 | XM_032744064.1 | XM_030466839.1 |
| ***LURAP1L*** | XM_027446757.1 | XM_001231706.5 | XM_031557285.1 | XM_015848999.2 | XM_030258345.2 | XM_030466745.1 |
| ***MPDZ*** | XM_027446716.1 | XM_025144729.1 | XM_031557296.1 | XM_032441256.1 | XM_030257311.1 | XM_030466746.1 |
| ***NFIB*** | XM_027446716.1 | XM_025144690.1 | XM_031557305.1 | XM_015849009.2 | XM_030258968.2 | XM_008496217.2 |
