# Supplementary material for: Transcriptome Analysis Reveals Genes Associated With Sexual Dichromatism of Head Feather Color in Mallard
Source: Front Genet. 2021 Dec 8;12:627974. doi: 10.3389/fgene.2021.627974 (PMC8692775; doi:10.3389/fgene.2021.627974)
Supplement: Supplementary file 3 [file Table9.DOCX]

**Table S9. Transcription factors predicted results of *TYR* and *TYRP1* promoters.**

| **Gene name/TF** | **Regulated gene** | **Source** | **Predict sequence** | **start** | **end** | **Strand** | **pvalue** | **Matched sequence** | **Male vs famale** | | **Head vs back** | |
| --- | --- | --- | --- | --- | --- | --- | --- | --- | --- | --- | --- | --- |
|  |  |  |  |  |  |  |  |  | **log2FoldChange** | **pvalue** | **log2FoldChange** | **pvalue** |
| ARX | *TYRP1* | database | NC_040075.1:30747598-30752598 | 5 | 17 | - | 1.57E-05 | TTAATTTTATTAG | 7.382374467 | 0.008307064 | 5.378874486 | 0.002423413 |
|  |  | database | NC_040075.1:30747598-30752598 | 5 | 17 | + | 8.64E-05 | CTAATAAAATTAA |  |  |  |  |
|  |  | database | NC_040075.1:30747598-30752598 | 1455 | 1467 | - | 3.39E-05 | ATAATTAGATTTG |  |  |  |  |
|  |  | database | NC_040075.1:30747598-30752598 | 1455 | 1467 | + | 5.02E-05 | CAAATCTAATTAT |  |  |  |  |
|  |  | database | NC_040075.1:30747598-30752598 | 4552 | 4564 | + | 1.27E-05 | TTTATTTGATTAT |  |  |  |  |
| MAFA | *TYRP1* | database | NC_040075.1:30747598-30752598 | 4238 | 4244 | - | 5.35E-05 | TCAGCAG | 3.12912248 | 0.01758499 | 4.594862812 | 0.00253497 |
|  | *TYR* | database | NC_040046.1:8264483-8269483 | 2044 | 2064 | - | 9.43E-06 | CTGCTGATGCTGAGGCAATCA |  |  |  |  |
|  |  | database | NC_040046.1:8264483-8269483 | 2044 | 2064 | - | 9.46E-06 | CTGCTGATGCTGAGGCAATCA |  |  |  |  |
|  |  | database | NC_040046.1:8264483-8269483 | 1273 | 1279 | + | 5.35E-05 | TCAGCAG |  |  |  |  |
|  |  | database | NC_040046.1:8264483-8269483 | 1509 | 1515 | + | 5.35E-05 | TCAGCAG |  |  |  |  |
|  |  | database | NC_040046.1:8264483-8269483 | 2058 | 2064 | + | 5.35E-05 | TCAGCAG |  |  |  |  |
|  |  | database | NC_040046.1:8264483-8269483 | 3099 | 3105 | + | 5.35E-05 | TCAGCAG |  |  |  |  |
|  |  | database | NC_040046.1:8264483-8269483 | 3894 | 3900 | + | 5.35E-05 | TCAGCAG |  |  |  |  |
|  |  | database | NC_040046.1:8264483-8269483 | 3869 | 3875 | - | 5.35E-05 | TCAGCAG |  |  |  |  |
|  |  | database | NC_040046.1:8264483-8269483 | 4337 | 4343 | - | 5.35E-05 | TCAGCAG |  |  |  |  |
|  |  | database | NC_040046.1:8264483-8269483 | 1495 | 1515 | - | 7.47E-05 | CTGCTGAACTTGCATTTCAGG |  |  |  |  |
|  |  | database | NC_040046.1:8264483-8269483 | 1495 | 1515 | - | 7.58E-05 | CTGCTGAACTTGCATTTCAGG |  |  |  |  |
| GSC | *TYR* | database | NC_040046.1:8264483-8269483 | 2748 | 2757 | + | 2.62E-05 | GCTAATCCAC | - | - | 2.280192649 | 0.000177104 |
| OTX1 | *TYR* | database | NC_040046.1:8264483-8269483 | 211 | 218 | - | 3.58E-05 | AGGATTAC |  |  | 2.688010706 | 6.25E-07 |
|  |  | database | NC_040046.1:8264483-8269483 | 211 | 218 | + | 3.58E-05 | GTAATCCT |  |  |  |  |
|  |  | database | NC_040046.1:8264483-8269483 | 3825 | 3832 | + | 5.75E-05 | AGGATTAA |  |  |  |  |
|  |  | database | NC_040046.1:8264483-8269483 | 3825 | 3832 | - | 5.75E-05 | TTAATCCT |  |  |  |  |
| FOXF2 | *TYRP1* | database | NC_040075.1:30747598-30752598 | 3824 | 3837 | + | 5.01E-05 | GAAAAGTAAATAAA | - | - | 2.276962293 | 0.014386772 |
|  |  | database | NC_040075.1:30747598-30752598 | 3825 | 3835 | - | 2.19E-05 | TATTTACTTTT |  |  |  |  |
|  |  | database | NC_040075.1:30747598-30752598 | 3825 | 3835 | + | 2.19E-05 | AAAAGTAAATA |  |  |  |  |
|  |  | database | NC_040075.1:30747598-30752598 | 4565 | 4575 | - | 8.64E-05 | TATTTGCTTTA |  |  |  |  |
|  |  | database | NC_040075.1:30747598-30752598 | 4565 | 4575 | + | 8.65E-05 | TAAAGCAAATA |  |  |  |  |
|  | *TYR* | database | NC_040046.1:8264483-8269483 | 4620 | 4630 | + | 9.93E-06 | TGTTTGCCTTT |  |  |  |  |
|  |  | database | NC_040046.1:8264483-8269483 | 4620 | 4630 | - | 9.93E-06 | AAAGGCAAACA |  |  |  |  |
