# Supplementary material for: Transcriptome Analysis Reveals Genes Associated With Sexual Dichromatism of Head Feather Color in Mallard
Source: Front Genet. 2021 Dec 8;12:627974. doi: 10.3389/fgene.2021.627974 (PMC8692775; doi:10.3389/fgene.2021.627974)
Supplement: Supplementary file 5 [file Table2.DOCX]

| **RNA-Seq sample** | **7th week male duck head skin （rep1）** | **7th week male duck head skin （rep2）** | **7th week male duck head skin （rep3）** | **11th week male duck head skin（rep1）** | **11th week male duck head skin（rep2）** | **11th week male duck head skin（rep3）** | **14th week male duck head skin（rep1）** | **14th week male duck head skin（rep2）** | **14th week male duck head skin（rep3）** | **14th week famale duck head skin（rep1）** | **14th week famale duck head skin（rep2）** | **14th week famale duck head skin（rep3）** | **14th week male duck back skin（rep1)** | **14th week male duck back skin（rep2)** | **14th week male duck back skin（rep3)** |
| --- | --- | --- | --- | --- | --- | --- | --- | --- | --- | --- | --- | --- | --- | --- | --- |
| **Clean reads** | 44050661 | 42495381 | 41453699 | 39032480 | 42374330 | 40502289 | 57230183 | 48603462 | 48359009 | 42522598 | 46547793 | 53182401 | 43872326 | 53424736 | 46157468 |
| **mapped reads** | 40917138 | 39922658 | 38484780 | 36439618 | 39620394 | 37542014 | 53677014 | 45326106 | 45034268 | 39495034 | 43454408 | 49561622 | 41002178 | 49952488 | 43219934 |
| **Mapping to gene** | 16856 | 16021 | 15761 | 15915 | 16317 | 15290 | 15913 | 15582 | 16005 | 15745 | 15977 | 16232 | 16337 | 15887 | 15910 |
| **Singletons** | 2861211 | 2362381 | 2608813 | 2360848 | 2562756 | 2625619 | 3358863 | 3085514 | 3058533 | 2692730 | 2949299 | 3387975 | 2659774 | 3321186 | 2831936 |

**Table S2. RNA-Seq data summary and annotation results.**
