# Supplementary material for: Transcriptome Analysis Reveals Genes Associated With Sexual Dichromatism of Head Feather Color in Mallard
Source: Front Genet. 2021 Dec 8;12:627974. doi: 10.3389/fgene.2021.627974 (PMC8692775; doi:10.3389/fgene.2021.627974)
Supplement: Supplementary file 10 [file Table10.DOCX]

**Table S10. The common pigmentation genes linked with Z-chromosomes.**

| **Gene name** | **Chr.** | **The detail locations** | | | | | | **Function** |
| --- | --- | --- | --- | --- | --- | --- | --- | --- |
|  |  | **Mallard** | **Chicken** | **Turkey** | **Japanese quail** | **Zebra finch** | **Anna's hummingbird** |  |
| ***MLANA*** | Z | 27924133-27929401 | 27814460-  27819704 | 25983291-  25989235 | 25038643-  25043318 | 25983291-  25989235 | 46198033-  46203316 | Maintain the stability of melanosome structure |
| ***SLC45A2*** | Z | 10921980-10936220 | 10331208-  10346018 | 9177933-  9194844 | 9487489-  9500745 | 9177933-  9194844 | 12555294-  12569214 | Maintain environmental homeostasis in melanin cells |
| ***TYRP1*** | Z | 10921980-10936220 | 30823102-  30833822 | 28188081-  28199921 | 27668114-  27687084 | 70260748-  70271371 | 43496550-  43507229 | Related to melanin biosynthesis |
